# Modeling of Multi-Cell HBT Device Based on Device Structure

**DOI:** 10.3390/mi16040433

**Published:** 2025-04-02

**Authors:** Haoyi Zhao, Jun Liu, Tao Rong, Shiyue Fan, Zhanfei Chen, Junchao Wang

**Affiliations:** 1 Innovation Center for Electronic Design Automation Technology, Hangzhou Dianzi University, Hangzhou 310018, China; 221040024@hdu.edu.cn (H.Z.);; 2 Ningbo Institute of Digital Twin, Eastern Institute of Technology, Ningbo 315200, China

**Keywords:** multi-cell device, HBT device modeling, radio frequency parasitic extraction, direct current characteristics, radio frequency characteristics, large-signal verification

## Abstract

This paper focuses on the modeling challenges of a multi-cell heterojunction bipolar transistor (HBT) used in radio frequency (RF) power amplifiers and proposes an innovative linear small-signal modeling method. Based on devices with an emitter size of 3 μm × 40 μm × 2–6 (emitter width × emitter length × emitter index-cell number), an equivalent circuit model including peripheral parasitic parameters is constructed by analyzing device layout characteristics in response to additional parasitic effects introduced by the multi-cell structure. A step-by-step parameter extraction method is used, with particular attention paid to the correction of saturated current parameters, temperature coefficients, thermal resistance correction, and the optimization of junction capacitance parameters based on the capacitance ratio relationship. After the extraction of parasitic parameters, the input and output reflection coefficient errors of the model under zero-bias conditions are below 1.66% in the 0.7–25 GHz frequency band. The accuracy of this model is significantly improved compared to the directly parallel single-cell model. The power simulation results match the measured results very well at frequencies of 2.6 GHz and 3.5 GHz. This modeling method significantly improves the model accuracy of multi-cell HBT devices in RF circuit design and provides an effective tool for high-power amplifier optimization.

## 1. Introduction

Heterojunction bipolar transistors (HBTs) possess the advantages of a high cutoff frequency, high gain, and high power density, and are widely used in radio frequency (RF) circuits, especially in power amplifiers [1,2,3,4,5,6]. In the design process of RF power amplifiers, in order to achieve better performance, it is usually necessary to conduct in-depth research and optimization of the circuit structure and the selected devices [7,8,9,10,11,12]. A multi-cell structure provides new ideas for the design of RF power amplifiers. In a multi-cell structure, each transistor operates at a lower voltage and reduced current, thereby enabling the handling of higher power levels. The lower power output of each transistor can reduce the nonlinear distortion of the transistors, and the even distribution of power can reduce the load on individual transistors. These advantages effectively improve the performance of the amplifiers [13].

Accurate device models can enhance the efficiency of circuit design and are used for analyzing the electromagnetic compatibility of RF circuits [14,15]. Multi-cell devices have many advantages as a circuit structure for high-power applications, but the literature on their models is less reported. Due to their compact structure, it is necessary to comprehensively extract a multi-cell device as a whole. At present, there is no mature model and extraction process that can be directly used for the modeling of multi-cell HBT devices. This paper innovatively proposes a modeling method for multi-cell HBT devices. In the modeling process, the peripheral parasitic effects introduced by the multi-cell structure are first considered, and an equivalent circuit of the multi-cell device model including the peripheral parasitic network is established. During the parameter extraction process, the impact of the multi-cell structure on the device output characteristics is given priority. The model is verified after parameter extraction. Ultimately, the model shows good linear small-signal fitting, and the power simulation results are basically consistent with the measured results. It is of great significance for guiding subsequent circuit design.

## 2. Methods for Device Model Construction, Extraction, and Optimization

### 2.1. Model Parameter Extraction Process

**Figure 1:**
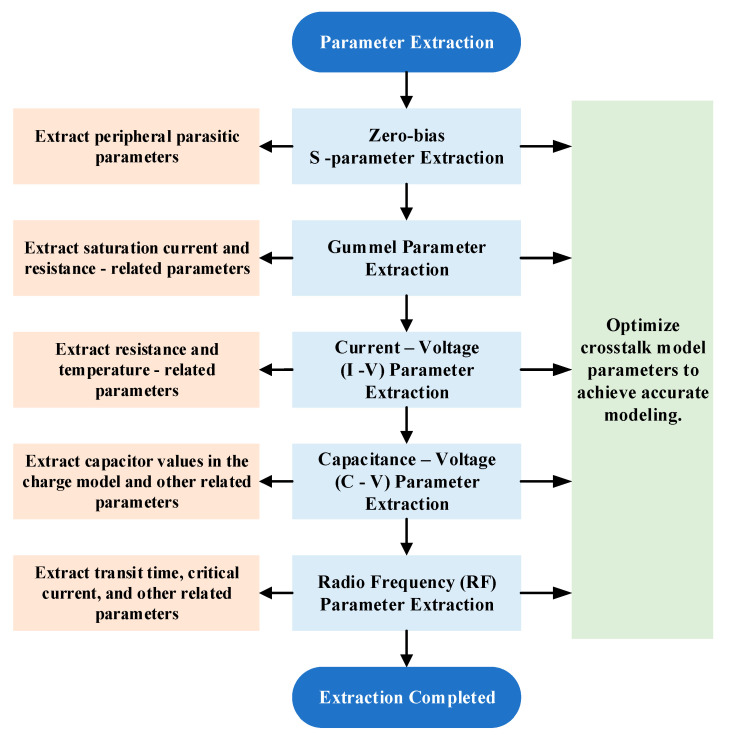
The modeling process of a multi-cell HBT device.

In this experiment, the size of the multi-cell HBT device used is 3 μm × 40 μm × 2–6 (emitter width × emitter length × emitter exponent-cell number), and the main operating frequency band is 0.7 to 25.1 GHz. The modeling process is shown in Figure 1. Compared with a single-cell HBT device, the structure of a multi-cell HBT device is more complex and compact, introducing additional parasitic effects, and exhibiting more pronounced coupling and thermal effects during device operation. Therefore, when modeling a multi-cell device, it is not possible to simply parallel the model of a single-cell device. Instead, based on the single-cell device model, comprehensive parameter extraction and optimization should be carried out by combining actual structure and measurement data [16].

### 2.2. Peripheral Parasitic Extraction for a Multi-Cell Device

**Figure 2:**
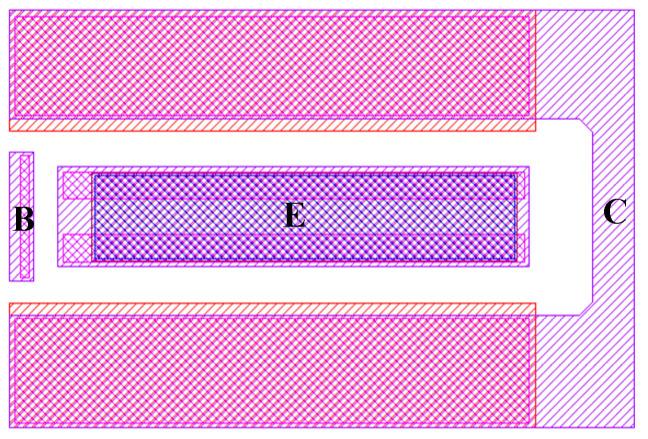
Single-cell HBT device layout structure.

**Figure 3:**
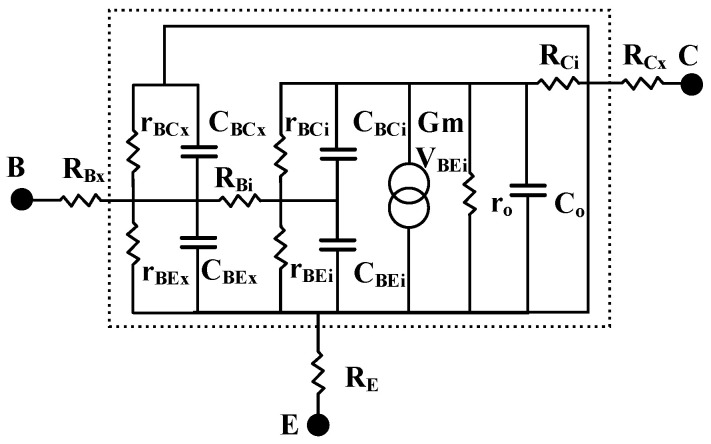
The small-signal equivalent circuit of a single-cell HBT device.

Referring to the parameter extraction process in Figure 1, the first step is to extract the peripheral parasitic parameters of the multi-cell device. Figure 2 and Figure 3 illustrate the layout structure of the single-cell device and the small-signal equivalent circuit model of the single-cell device used in the experiment. The intrinsic part of the single-cell device is enclosed within the dashed lines, while the parasitic resistances at the base, collector, and emitter ports of the single-cell device are denoted as *R_Bx_*, *R_Cx_*, and *R_E_*, respectively, outside the dashed lines.

**Figure 4:**
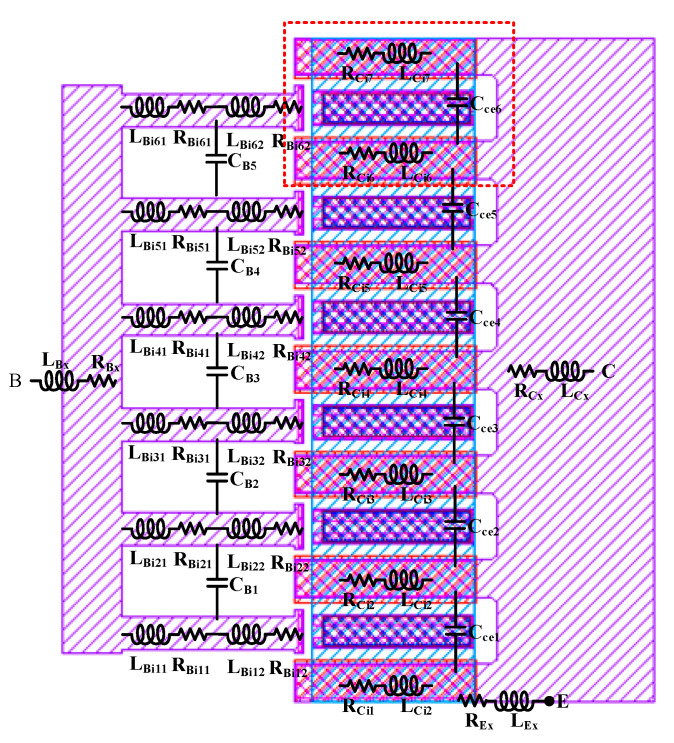
The layout structure of a multi-cell HBT device.

**Figure 5:**
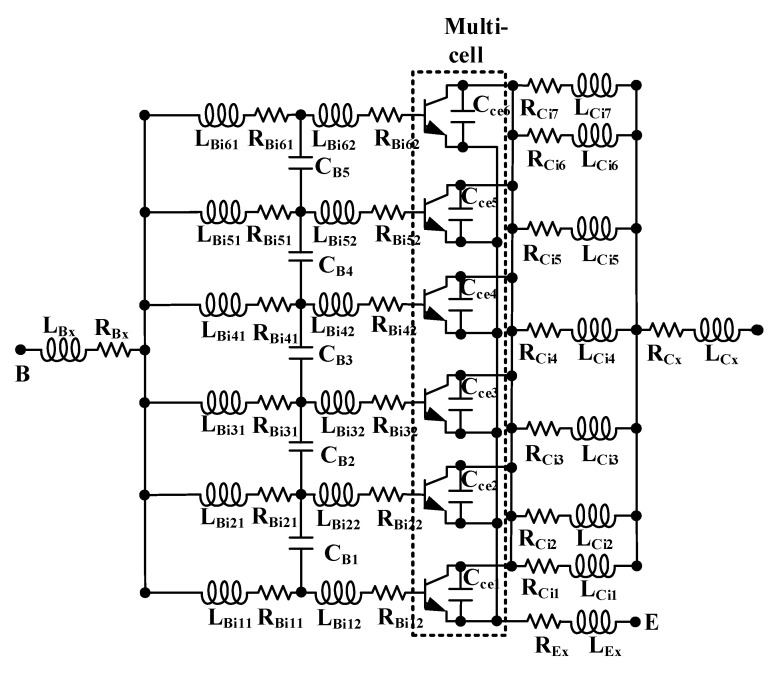
The equivalent circuit of the multi-cell HBT device.

Figure 4 illustrates the layout structure of a multi-cell HBT device, with the dashed box indicating the single-cell section. An analysis of the layout structure indicates that, compared to single-cell devices, multi-cell devices introduce additional parasitic elements, including the following: the parasitics of the lumped base, emitter, and collector metal leads; the coupling capacitance between the lumped emitter metal leads and the collectors of each cell; the coupling capacitance between the base leads of each cell; and the additional parasitics generated by the high-frequency distribution effects among the leads of each cell under high-frequency conditions [17,18,19]. Based on the aforementioned analysis, the equivalent circuit of the multi-cell HBT device as illustrated in Figure 5 has been established. The parasitic effects of the lumped base, collector, and emitter leads are represented by *L_Bx_*, *R_Bx_*, *L_Ex_*, *R_Ex_*, *L_Cx_*, and *R_Cx_*. The coupling capacitance between the base leads is characterized by capacitors *C_Bi_*_(*i*=1–5)_, while the coupling capacitance between the lumped emitter and the collectors of each cell is denoted by capacitors *C_cei_*_(*i*=1–6)_. The high-frequency distribution effects of the base leads of each cell are represented by *L_Binm_*_(*n*=1–6; *m*=1–2)_ and *R_Binm_*_(*n*=1–6; *m*=1–2)_, and those of the collector leads of each cell are represented by *L_Cinm(n_*_=1–7; *m*=1–2)_ and *R_Cinm_*_(*n*=1–7; *m*=1–2)_. The locations of these parasitic elements are annotated in the layout structure.

In the above parasitic, the coupling capacitance is small, which can be directly obtained by using the parallel plate capacitor calculation formula:

(1) $$C=\frac{\varepsilon_{0}\varepsilon_{r}A}{d}$$

In Formula (1), *ε*_0_ represents vacuum permittivity, and ε*_r_* represents the relative permittivity of the material. *A* represents the relative area of the two metal plates, and *d* is the distance between the metal plates.

**Figure 6:**
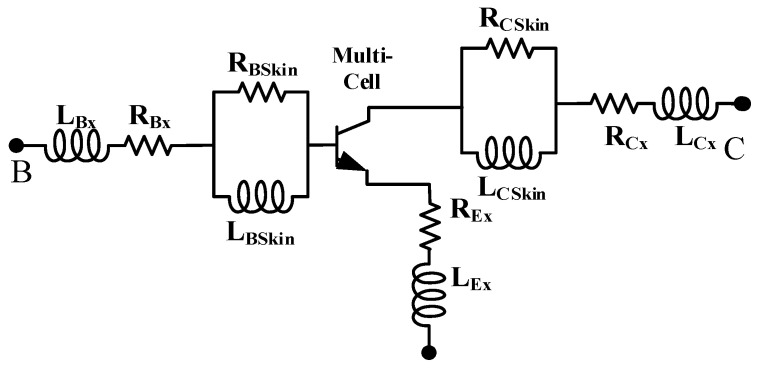
Simplified diagram of equivalent circuit of multicellular device.

In order to simplify the parameter extraction process, the equivalent circuit in Figure 5 is simplified to the style in Figure 6. *R_BSkin_*, *L_BSkin_*, *R_CSkin_*, and *L_CSkin_* are used to characterize the high-frequency distribution effect. The multi-cell represents the part within the dotted line in Figure 5.

At low frequencies (*f* < 1 GHz), the parasitic effects of the metal interconnects in each cell are relatively small, and *R_Bskin_*, *L_BSkin_*, *R_CSkin_*, and *L_CSkin_* can be considered as short circuits. Therefore, at low frequencies, it is only necessary to consider the parasitic interconnects of the lumped base, collector, and emitter, namely *L_Bx_*, *R_Bx_*, *L_Ex_*, *R_Ex_*, *L_Cx_*, and *R_Cx_*.

The Z-parameter expression for the equivalent circuit under low-frequency conditions is established:

(2) $$Z_{\mathrm{total}}=\left[Z_{X}+Z_{mult}\right]$$

In Formula (2), *Z_total_* is obtained by converting the scattering parameters measured under low-frequency conditions through a matrix transformation formula. *Z_mult_* is derived from the parallel connection of the port network matrices of the single-cell devices. *Z_X_* represents the port network of the interconnect parasitic effects as follows:

(3) $$Z_{X}=\left[\begin{array}{cc} \left(R_{Bx}+L_{Bx}\right)+\left(R_{Ex}+L_{Ex}\right) & \left(R_{Ex}+L_{Ex}\right)\\ \left(R_{Ex}+L_{Ex}\right) & \left(R_{Cx}+L_{Cx}\right)+\left(R_{Ex}+L_{Ex}\right) \end{array}\right]$$

(4) $$Z_{X11}=R_{Bx}+j\omega L_{Bx}+R_{Ex}+j\omega L_{Ex}$$

(5) $$Z_{X22}=R_{Cx}+j\omega L_{Cx}+R_{Ex}+j\omega L_{Ex}$$

(6) $$Z_{X12}=Z_{X21}=R_{Ex}+j\omega L_{Ex}$$

In the formulas, the resistances (*R*) and inductances (*L*) correspond to the values of the individual components in the equivalent circuit, where *j* denotes the imaginary unit, and ω represents the angular frequency.

From Formulas (4)–(6), the following can be inferred:

(7) $$R_{Ex}=real(Z_{X21})$$

(8) $$L_{Ex}=imag(Z_{X21})$$

(9) $$R_{Bx}=real(Z_{X11}-Z_{X21})$$

(10) $$L_{Bx}=imag(Z_{X11}-Z_{X21})$$

(11) $$R_{Cx}=imag(Z_{X22}-Z_{X21})$$

(12) $$L_{Cx}=imag(Z_{X22}-Z_{X21})$$

At this point, the parasitic interconnects of the lumped part are extracted under low-frequency conditions. As the frequency increases, the parasitic effects of the metal interconnects in each cell become more pronounced due to high-frequency distribution effects and cannot be neglected. Therefore, the port network for the parasitic effects is revised as follows [20]:

(13) $$Z_{X\_All}=\left[\begin{array}{cc} Z_{X11}+\left(R_{BSkin}∥L_{BSkin}\right) & Z_{X12}\\ Z_{X21} & Z_{X22}+\left(R_{CSkin}∥L_{CSkin}\right) \end{array}\right]$$

(14) $$Z_{X\_All11}=R_{Bx}+j\omega L_{Bx}+R_{Ex}+j\omega L_{Ex}+\frac{R_{BSkin}j\omega L_{BSkin}}{R_{BSkin}+j\omega L_{BSkin}}$$

(15) $$Z_{X\_All22}=R_{Cx}+j\omega L_{Cx}+R_{Ex}+j\omega L_{Ex}+\frac{j\omega R_{CSkin}L_{CSkin}}{R_{CSkin}+j\omega L_{CSkin}}$$

By simultaneously considering Formulas (6), (14) and (15), we arrive at the following:

(16) $$Z_{X\_All11}-Z_{X12}=\left(R_{Bx}+\frac{\omega^{2}L_{BSkin}^{2}R_{BSkin}}{R_{BSkin}^{2}+\omega^{2}L_{BSkin}^{2}}\right)+j\omega \left(L_{Bx}+\frac{L_{BSkin}B_{BSkin}^{2}}{R_{BSkin}^{2}+\omega^{2}L_{BSkin}^{2}}\right)$$

(17) $$Z_{X\_All22}-Z_{X12}=\left(R_{Cx}+\frac{\omega^{2}L_{CSkin}^{2}R_{CSkin}}{R_{CSkin}^{2}+\omega^{2}L_{CSkin}^{2}}\right)+j\omega \left(L_{Cx}+\frac{L_{CSkin}B_{CSkin}^{2}}{R_{CSkin}^{2}+\omega^{2}L_{CSkin}^{2}}\right)$$

By substituting the values of *L_Bx_*, *R_Bx_*, *L_Cx_*, and *R_Cx_* obtained under low-frequency conditions into Formulas (16) and (17), the values of *R_BSkin_*, *L_BSkin_*, *R_CSkin_*, and *L_CSkin_* can be derived from their real and imaginary components. Once the values of *R_BSkin_* and *L_BSkin_* are determined, the values of *L_Binm_*_(*n*=1–6; *m*=1–2)_ and *R_Binm_*_(*n*=1–6; *m*=1–2)_ can be directly calculated using Equation (18).

(18) $$R_{BSkin}+L_{BSkin}=R_{Bi11}+L_{Bi11}+R_{Bi12}+L_{Bi12}//\cdots //R_{Bi61}+L_{Bi61}+R_{Bi62}+L_{Bi62}$$

Since the base interconnects of each cell are identical, the values of *L_Binm_*_(*n*=1–6; *m*=1–2)_ and *R_Binm_*_(*n*=1–6; *m*=1–2)_ are the same. As can be observed from the layout structure in Figure 3, the multi-cell device is not simply a parallel connection of single-cell devices; the collector structures of the cells are shared. In this case, the component values representing the high-frequency distributed effects of the collectors cannot be simply averaged. The values of *L_Cinm_*_(*n*=1–7; *m*=1–2)_ and *R_Cinm_*_(*n*=1–7; *m*=1–2)_ are divided into two categories: the equivalent component values of the two collector interconnects located at the periphery of the multi-cell device are defined as *L_out_* and *R_out_*, while the equivalent component values of the five internal collector interconnects are defined as *L_mid_* and *R_mid_*. From Figure 4, it can be seen that the four internal cells share collectors with adjacent cells. Assuming that the collector currents of each cell are equal, the equivalent cross-sectional area of the shared collector interconnects is half that of the non-shared interconnects. This leads to the derivation of Formulas (19) and (20).

(19) $$R_{out}\approx \frac{1}{2}R_{mid},$$

(20) $$L_{out}\approx \frac{1}{2}L_{mid},$$

Once this ratio is obtained, the values of *L_Cinm_*_(*n*=1–7; *m*=1–2)_ and *R_Cinm_*_(*n*=1–7; *m*=1–2)_ can be determined using the method described in Formula (18).

### 2.3. Model Parameter Extraction and Optimization for a Multi-Cell Device

After the extraction of peripheral parasitic parameters of the multi-cell device is completed, following the parameter extraction process, parameter optimization for the multi-cell device based on the parameters of the single-cell device model and measurement data is carried out.

1. Gummel parameter extraction

During the Gummel parameter extraction process, it is possible to determine the parameters related to the saturation current of the diodes in the HBT model, and the resistance parameters in the model can be extracted based on the high-bias voltage region.

In the multi-cell structure, due to the compact structure, the current coupling effect between the cells is significant [21]. Therefore, the total output current of a multi-cell device is less than that of the equivalent number of single-cell devices in parallel. During the Gummel parameter extraction process, it is necessary to reduce the parameters related to the saturation current to achieve a higher fitting accuracy.

2. Current-voltage parameter extraction

In the device’s *I_c_*-*V_c_* curve, parameters such as resistance and its temperature coefficient are mainly extracted. The emitter of a multi-cell device heats up more significantly than that of a single-cell device, and the self-heating effect is more pronounced. Therefore, when extracting current-voltage parameters, it is necessary to focus on the temperature coefficient and the thermal resistance of the device [22,23,24].

3. Capacitance-voltage parameter extraction

With variations in the bias voltage, capacitance can be categorized into three distinct regions: complete depletion, partial depletion, and forward bias. The built-in potential can be determined from the fully depleted region, while the zero-bias junction capacitance value and capacitance grading factor can be obtained from the partially depleted region.

**Figure 7:**
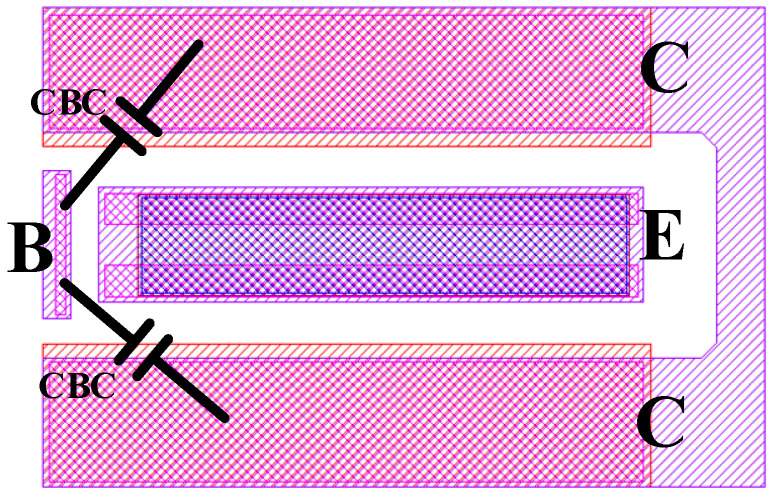
Schematic diagram of collector junction capacitance.

Due to the collector multiplexing of the internal cells in a multi-cell device, the zero-bias collector junction capacitance of each cell can no longer simply use the same value. Figure 7 annotates the location of collector junction capacitance generation in the actual layout structure of a single-cell device.

**Figure 8:**
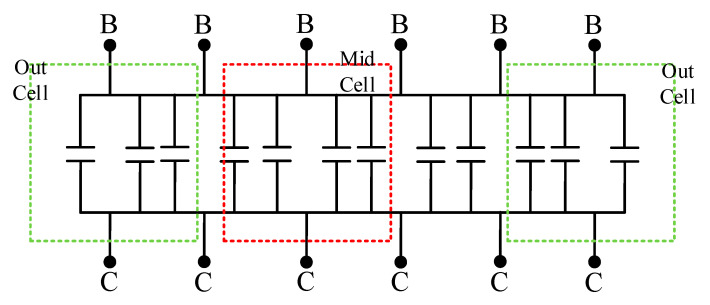
Equivalent schematic diagram of the zero-bias collector junction capacitance of a multi-cell device.

Figure 8 is an equivalent schematic diagram of the zero-bias collector junction capacitance of a multi-cell device established according to Figure 4 and Figure 7.

Since the collector areas in a multi-cell device are all the same, the capacitance values in the figure are equal and set to *C_BCi_*. The blue dashed line box selects the two outermost cells of the multi-cell device, and the green dashed line box selects an internal cell of the multi-cell device. The zero-bias junction capacitance of the two outermost cells is defined as *C_BCout_*, and the zero-bias junction capacitance of the internal cell is defined as *C_BCmid_*. According to the capacitor parallel relationship, the following can be concluded:

(21) $$C_{BCout}=3\times C_{BCi}$$

(22) $$C_{BCmid}=4\times C_{BCi}$$

(23) $$C_{BCout}=\frac{3}{4}\times C_{BCmid}$$

The ratio in Formula (23) was referred to in order to determine the zero-bias collector junction capacitance value of each cell in a multi-cell device during the capacitance-voltage parameter extraction process.

4. RF parameter extraction

Accurate DC and CV extraction is the foundation of RF extraction. In addition to the impact of DC and CV components, the RF characteristics of a device are mainly related to carrier transit time and the Kirk effect. When the output current is low, the carrier transit time is mainly concentrated in the collector region. When the device enters a high-current state, the Kirk effect begins to dominate. At this point, parameter extraction needs to comprehensively consider the DC and CV characteristics of the device and continuously iterate to ultimately obtain a good fitting result.

### 2.4. Multi-Cell Large-Signal Verification

**Figure 9:**
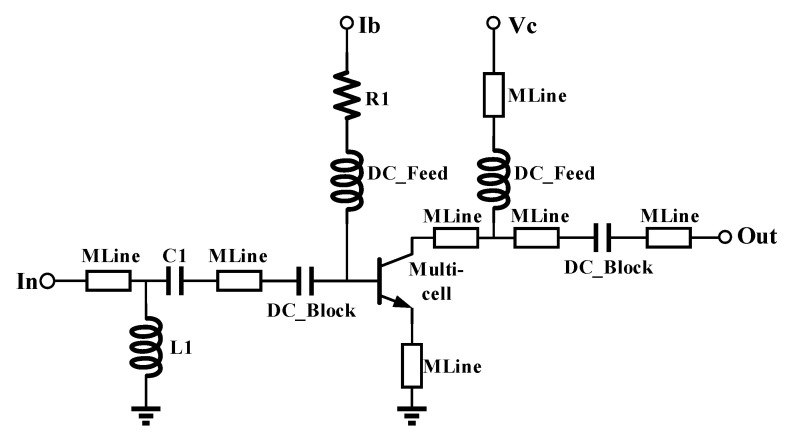
Power simulation circuit diagram.

After the extraction of the small-signal model parameters, the model was applied to the measurement circuit. The input load was set to 50 ohms, and the output load was set to the optimal load impedance obtained using the load-pull measurement method. Figure 9 shows the schematic diagram of the measurement circuit. In the schematic, the *MLine* is used to represent the microstrip line structure. The capacitor *C*1 and the inductor *L*1 are used to increase the impedance, enabling the device to operate safely under the test conditions. The ideal resistor *R*1 in the direct current bias branch is used to simulate the lead resistance value from the current mirror structure providing the direct current bias to the base of the multi-cell device in the actual circuit, which is given by the device designer. The *DC_Feed* and *DC_Block* components are used to block the alternating current and direct current signals in the simulation circuit.

The circuit in Figure 9 was processed to obtain the fabricated device. Load-pull measurements were performed on the circuit at 2.6 GHz and 3.5 GHz as required by the circuit design engineer. The obtained impedance values were used as conditions for power simulation. Based on the differences between the simulation results and the measurement results, and under the premise of ensuring the fitting accuracy of the small-signal model, the model parameters were fine-tuned. Ultimately, a multicellular device model with high accuracy under both large-signal and small-signal operating conditions was obtained.

## 3. Experiment and Results

The model parameters for six-cell devices have been extracted using the outlined method and fitted in IC-CAP 2018 using the Hepeesofsim emulator.

The specific values of the external parasitic parameters obtained through the extraction algorithm are presented in Table 1.

**Figure 10:**
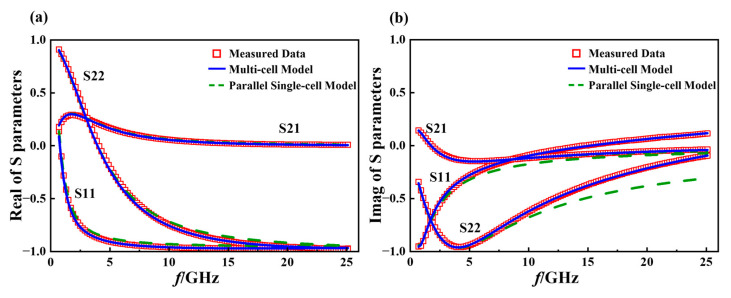
Zero-bias S-parameter fitting results and model comparison in the frequency range of 0.7–25.1 GHz. (**a**) Real component of S-parameters; (**b**) imaginary component of S-parameters.

Figure 10 shows a comparison of the S-parameter characteristics between the model after parasitic extraction and the model after directly paralleling single-cell devices under zero bias and a frequency range of 0.7–25.1 GHz. It can be seen from the figure that after extracting the peripheral parasitic parameters, the fitting accuracy of the reflection coefficient of the device is significantly increased.

To better demonstrate the effectiveness of the extraction method and evaluate model fitting accuracy, the following error formula was introduced [25]:

(24) $$e=\frac{\left|S_{sim(i)}-\left.S_{mea(i)}\right|\right.}{\sqrt{\frac{\sum_{i=1}^{n}{\left[S_{mea(i)}\right]}^{2}}{n}}}$$

In Formula (24), *e* represents the error value; *S_sim_*_(*i*)_ is the *i*-th simulation data; *S_mea_*_(*i*)_ is the *i*-th measured data; and *n* is the number of data points.

Table 2 presents the simulation and measured S parameter errors for two types of models: one is the multi-cell device model after extracting external parasitic parameters, and the other model is obtained by directly paralleling six single-cell device models sized 3 μm × 40 μm × 2, forming a multi-cell device model. In combination with Figure 10, it demonstrates the accuracy and effectiveness of the extraction of external parasitic parameters.

The fitting results of the multi-cell device model after parameter optimization are presented below, and a comparison was made with the simulated results of directly paralleling single-cell devices.

**Figure 11:**
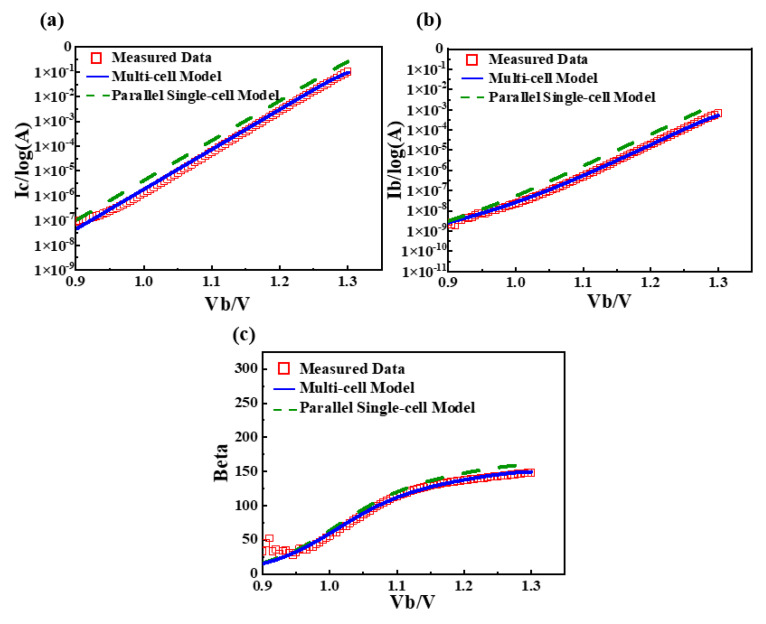
Gummel plot fitting results and model comparison. (**a**) *I_b_*-*V_b_* curve; (**b**) *I_c_*-*V_b_* curve; (**c**) *Beta*-*V_b_* curve.

The measurement conditions for Gummel parameter extraction are as follows: *V_c_* = *V_b_* within 0.7~1.3 V, *V_e_* = 0 V. Gummel curve fitting results are shown in Figure 11.

By focusing on the saturation current parameter, the Gummel plot achieves a close fit, resulting in a significant enhancement in the accuracy of the current gain *Beta*.

**Figure 12:**
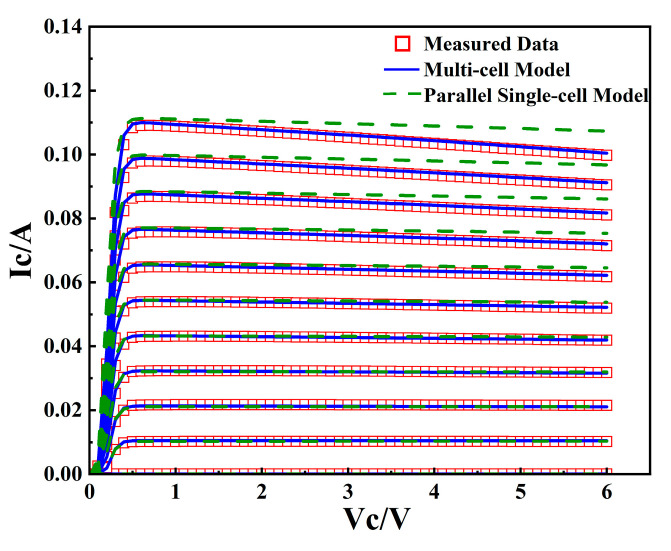
Fitting result and model comparison of *I_c_*-*V_c_* curve.

The measurement conditions for current-voltage parameter extraction are as follows: *I_b_*: 0~720 μA, *V_c_*: 0~6 V, *V_e_* = 0 V. Current-voltage curve fitting results are shown in Figure 12.

As shown in the figure, by prioritizing the optimization of the model’s temperature coefficient and thermal resistance, the fitting accuracy of the *I_c_*-*V_c_* curve, particularly in the high collector voltage regions, has been significantly improved.

**Figure 13:**
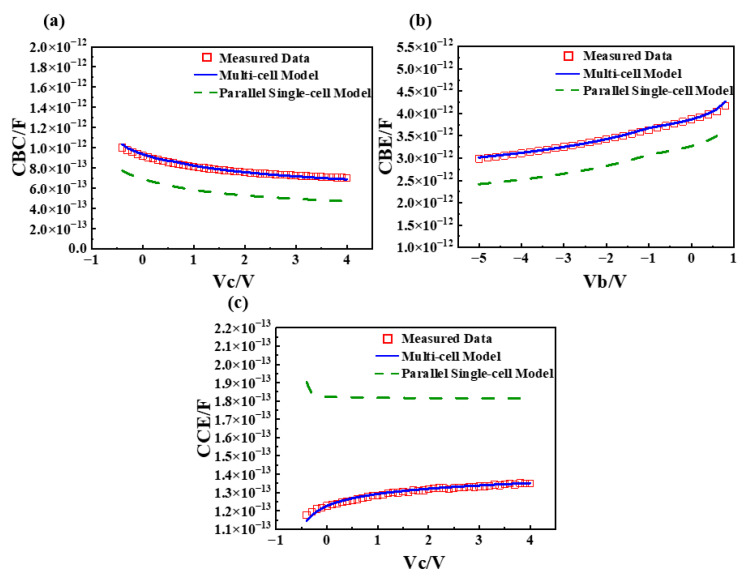
Capacitance-voltage characteristic fitting results and model comparison. (**a**) *CBC*-*V_c_* curve; (**b**) *CBE*-*V_b_* curve; (**c**) *CCE*-*V_c_* curve.

The measurement conditions for capacitance-voltage parameter extraction are as follows: CBE: *freq* = 3.5 GHz, *V_c_* = *V_e_* = 0 V, *V_b_* = −5~0.8 V; CBC: *freq* = 3.5 GHz, *V_b_* = *V_e_* = 0 V, *V_c_* = −0.4~4 V; CCE: *freq* = 3.5 GHz, *V_b_* = *V_e_* = 0 V, *V_c_* = −0.4~4 V. Capacitance-voltage curve fitting results are shown in Figure 13.

By adopting a proportional allocation method for device capacitance, improved accuracy in capacitance-voltage curve fitting was achieved, thereby laying the foundation for the modeling of radio frequency characteristics.

**Figure 14:**
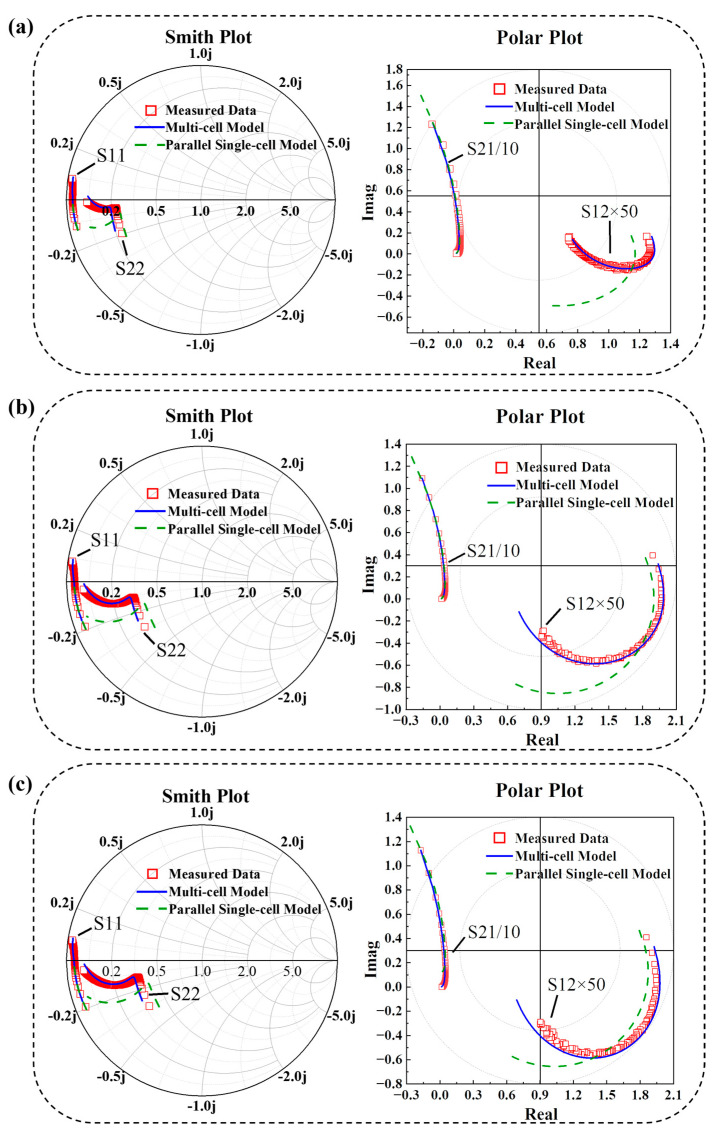
RF condition S-parameter fitting results and model comparison under the conditions of (**a**) *I_b_* = 288 μA, *V_c_* = 3 V; (**b**) *I_b_* = 144 μA, *V_c_* = 3 V; (**c**) *I_b_* = 144 μA, *V_c_* = 4 V.

Referring to the bias of the power circuit, the measurement conditions selected for RF fitting of the multi-cell HBT device are as follows: *V_c_*: 0~6 V, *I_b_:* 0~720 μA, *freq*: 0.7~25.1 GHz. Figure 14 presents a comparison between simulated and measured S-parameters under three bias conditions: (a) *I_b_* = 288 μA, *V_c_* = 3 V; (b) *I_b_* = 144 μA, *V_c_* = 3 V; (c) *I_b_* = 144 μA, *V_c_* = 4 V.

The results above demonstrate the effectiveness of the parameter extraction method used in this experiment.

**Figure 15:**
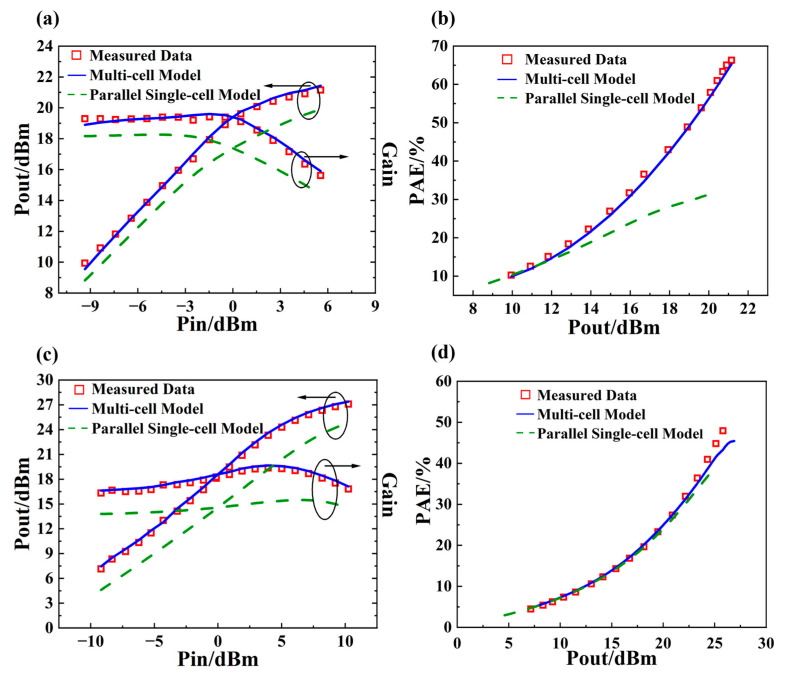
Fitting results and model comparison. (**a**) *Pout*/*Gain*-*Pin* curve at 2.6 GHz; (**b**) *PAE*-*Pout* curve at 2.6 GHz; (**c**) *Pout*/*Gain*-*Pin* curve at 3.5 GHz; (**d**) *PAE*-*Pout* curve at 3.5 GHz.

To further validate the reliability and accuracy of the extracted model, the optimized device is integrated with the electromagnetic (EM) structure of the actual circuit in ADS 2017 software for co-simulation. Under the operating frequency of 2.6 GHz, the bias conditions are set as *I_b_* = 187 μA and *V_c_* = 3.4 V, with a load impedance of 23.87 + 29.94*j ohm*. At 3.5 GHz, the bias conditions remain *I_b_* = 187 μA and *V_c_* = 3.4 V, while the load impedance is adjusted to 8.552 + 2.101*j ohm*. Figure 15 shows the fitting and comparison results of the output power and power added efficiency at the corresponding frequency points.

At 2.6 GHz, the output power error is within 1.2 dBm, and the power added efficiency curve shows the correct trend, and the error is within 2.6%. At 3.5 GHz, the output power error is within 0.9 dBm, and the power added efficiency error is within 4.8%. These results prove the reliability and accuracy of the extracted model.

## 4. Conclusions

This study proposes a refined modeling methodology for multi-cell heterojunction bipolar transistor (HBT) devices. Experiments were performed based on a multi-cell device with a size of 3 μm × 40 μm × 2–6. In terms of equivalent circuit modeling, a precision small-signal equivalent circuit topology was constructed based on actual device layout characteristics, accompanied by the development of a parasitic parameter extraction algorithm. Experimental results demonstrate that compared with the conventional parallelization approach of single-cell devices with a size of 3 μm × 40 μm × 2, the proposed model achieves an average 11.84% improvement in S-parameter fitting accuracy across 0.7–25.1 GHz under zero-bias conditions. Notably, the S11 and S22 fitting accuracies reach 98.63% and 98.34%, respectively, significantly enhancing model-measurement consistency. Special considerations were given to device coupling effects, self-heating phenomena, and capacitance distribution challenges arising from collector terminal sharing during modeling. To validate engineering applicability, system-level verification was conducted through high-power RF amplifier circuits. The results show that at 2.6 GHz and 3.5 GHz, the output power error is less than 1.2 dBm, and the power added efficiency error is less than 4.8%, confirming model reliability under large-signal excitation. It should be noted that constrained by current process limitations, protective measurement strategies were implemented for specific parameters. Future work will focus on the co-optimization of device architecture and fabrication processes, while establishing a multi-dimensional validation framework extending to millimeter-wave frequencies, thereby providing a more precise simulation foundation for high-power RF integrated circuit design.

## Figures and Tables

**Table 1:** Extraction results of external parasitic parameters.

Parameter	Values
*R_Bx_* (Ω)	0.119
*L_Bx_* (pH)	27.86
*R_Cx_* (Ω)	0.0158
*L_Cx_* (pH)	30.9
*R_Ex_* (Ω)	0.0997
*L_Ex_* (pH)	2.66
*C_Bn_*_(*n*=1–5)_ (fF)	0.158
*C_cen_*_(*n*=1–6)_ (fF)	21.8
*R_Binm_*_(*n*=1–6; *m*=1–2)_ (Ω)	0.455
*L_Binm_*_(*n*=1–6; *m*=1–2)_ (pH)	3.97
*R_Cin_*_(*n*=2–5)_ (Ω)	0.069
*L_Cin_*_(*n*=2–5)_ (pH)	18.82
*R_Cin_*_(*n*=1; 6)_ (Ω)	0.0345
*L_Cin_*_(*n*=1; 6)_ (pH)	9.41

**Table 2:** Comparison of model S parameter errors.

Model	S11	S22	S21	S12
Extracting external parasitic	1.37%	1.66%	5.17%	5.17%
Directly paralleling	12.11%	12.63%	17.99%	18.00%

## Data Availability

The datasets presented in this article are not readily available because the data are part of an ongoing study. Requests to access the datasets should be directed to 221040024@hdu.edu.cn.
